# Schwann Cells and Their Exosomes: Research Progress and Prospect in Spinal Cord Injury

**DOI:** 10.1155/np/6684089

**Published:** 2025-06-12

**Authors:** Xin Wang, Wei Yan, Lin Zhu, Lingzhi Wei, Haobo Cao, Fanni Yang, Yibao Zhang

**Affiliations:** ^1^The Second Hospital and Clinical Medical School, Lanzhou University, Lanzhou, Gansu Province, China; ^2^Hospital of Stomatology Lanzhou University, Lanzhou University, Lanzhou, Gansu Province, China

**Keywords:** cell transplantation, exosomes, Schwann cells, spinal cord injury

## Abstract

Spinal cord injury (SCI) is a severe condition that affects the central nervous system (CNS), for which there is currently no effective treatment. Schwann cells (SCs) transplantation for SCI has been well demonstrated in preclinical studies, showing that it can achieve therapeutic goals by improving autonomic function, reducing neuropathic pain, and enhancing limb function through mechanisms such as alleviating inflammation, modulating immunity, and reducing dense scar formation. However, the transplantation of SCs sometimes encounters adverse events, such as low survival rates, significant rejection reactions, limitations on transplantation methods, and the formation of glial scars, all of which severely hinder its clinical application. Meanwhile, SC-derived exosomes (SC-exos) also hold great potential in treating SCI, with specific roles, including immune modulation, anti-inflammatory effects, angiogenesis, apoptosis inhibition, and promotion of axonal regeneration, even surpassing traditional cell therapy in certain aspects. This paper aims to elucidate the potential mechanisms and valuable therapeutic roles of SCs and SC-exos in the treatment of SCI, as well as to provide insights for subsequent research directions by analyzing their current limitations.

## 1. Introduction

Spinal cord injury (SCI) is a debilitating condition affecting the central nervous system (CNS) and can arise from various causes. Clinically, SCI is characterized by impairments in motor and sensory functions below the level of the injury. There are two primary categories of SCIs: traumatic and nontraumatic. Traumatic SCIs result from external forces, including sports injuries, traffic accidents, falls, and physical assaults [1]. Nontraumatic SCIs, in contrast, arise from factors such as ischemia, infection, spinal cord tumors, inflammation, and neurodegenerative diseases [2, 3]. Both types of SCIs can have a profound impact on bodily functions.

The pathophysiological mechanisms of SCI are complex and can be categorized into two stages: primary injury and secondary injury [4]. Primary injury refers to the direct damage inflicted on neurons and/or blood vessels by external forces. This injury results in inflammatory extravasation, alterations in cell membrane permeability, and the activation of apoptotic signals [5]. Although the primary injury is not the most challenging stage of SCI, its severity can significantly influence disease outcomes and prognosis. Secondary injury is further categorized into three phases: the acute phase (days), the moderate phase (less than 14 days), and the chronic phase (weeks or months) [6]. These phases are characterized by various pathological processes, including inflammation, apoptosis, disruption of the blood–spinal cord barrier (BSCB), neuronal demyelination, glial scarring, vascular destruction, and deposition of chondroitin sulfate (CS) proteoglycans (CSPGs) [7–12].

Despite extensive research into the pathophysiological mechanisms underlying SCI, effective treatment options continue to be limited [13]. This treatment gap not only results in significant psychological and physical suffering for patients and their families, but it also places a substantial economic burden on society [14]. As a result, researchers in relevant fields worldwide are actively striving to explore effective methods for the prevention, treatment, and rehabilitation of SCI, as it has become a shared objective.

Schwann cells (SCs) are glial cells primarily located in the peripheral nervous system (PNS). They encase neuronal axons to form myelin sheaths, thereby nourishing, protecting, and facilitating the repair of neurons [15]. Initially believed to be present solely in the PNS, recent studies have identified the presence of SC in the CNS under certain conditions, including multiple sclerosis, neuromyelitis optica, SCI, and CNS precursor transplantation [16, 17]. This suggests a potential link between SCs and the CNS. However, it remains unconfirmed whether SCs originate from the CNS or migrate from the periphery across the BSCB under specific conditions.

Numerous studies have demonstrated that the central transplantation of SCs and their precursor cells can effectively treat SCI. Experimental animal models indicate that SCs transplantation can improve autonomic nervous system function, prevent pathological thickening of the bladder wall [18], reduce scar tissue formation [19], and enhance limb movement. Clinical trials further support the safety and efficacy of this approach in alleviating pain [20]. Although the precise mechanisms underlying these effects remain unclear, advancements in SCs transplantation technology are increasingly promising as a novel treatment for SCI.

Exosomes, which have a diameter of 30–200 nm, are extracellular vesicles originating from endosomes. They contain various types of RNAs (noncoding RNA, small interfering RNA, messenger RNA, ribosomal RNA, etc.), proteins, and specific lipids. These exosomes play a crucial role in intercellular communication [21]. Initially discovered in sheep reticulocytes in 1983, exosomes were initially believed to be involved in the secretion of metabolic waste. However, subsequent studies have revealed that exosomes can be derived from most cells. Although their contents do not directly participate in cell metabolism, they can indirectly influence cell fate [22–25]. It is important to note that while the process and structure of exosomes are generally similar, there are significant differences in the mechanism, content, and function of exosomes produced by different sources and in response to different stimuli [26].

Because of their small size, simple extraction, high stability, and ability to pass through the blood–brain barrier (BBB) and BSCB to reach the CNS [27], exosomes have gained increasing attention for their therapeutic potential in CNS diseases. Exosomes carry various biological macromolecules related to cell functions [28–30]. In particular, exosomes derived from mesenchymal stem cells have been found to promote axonal growth [31–33], vascular regeneration [34], and regulate inflammatory immune response [35, 36] and the complement system [37, 38]. They have also been shown to reduce the permeability of the BSCB [39], making them a promising method for treating SCI.

Additionally, exogenous SCs transplantation has been proven to have therapeutic effects on SCI [40]. SC-derived exosomes (SC-exos) have also been gradually recognized for their similar effects to mesenchymal stem cell-derived exosomes (MSC-exos), and in some aspects, they have shown even better results than MSC-exos. Therefore, this review aims to summarize the recent literature on SCs and SC-exos, elucidate the mechanisms of some SC-exos in SCI treatment, and propose unproven conjectures and potential based on existing research.

## 2. The Physiological Functions and Preliminary Application of SCs in SCI

SCs play a crucial role in the PNS by forming the myelin sheath around myelinated nerve fibers. This sheath enhances the conduction speed of electrical impulses along the neuron's axon, facilitating the propagation of signals. Additionally, SCs have various functions, such as cell regulation, differentiation, nerve repair, neurotrophy, and immune regulation (as shown in Table 1). Duncan et al. [61] discovered that the CNS is protected by a glial membrane composed of glial cells and nerve extracellular matrix, preventing the entry of external tissue cells. In cases of SCI, this membrane may be damaged, allowing endogenous to enter the CNS through the injury site [62]. On the other hand, SCs transplantation involves the injection of purified and identified into the affected area under specific conditions. Clinical studies have demonstrated the reliable and safe therapeutic effects of SCs transplantation [63–65].

### 2.1. SCs Can Improve Limb Function After SCI

Whether in experimental animals or humans, the most prominent feature following SCI is the partial or complete loss of limb function. Depending on the specific spinal segment that is injured, patients may exhibit dysfunction in either the upper or lower limbs. SCI not only directly damages the CNS but may also impact peripheral nerve function through various mechanisms, including nerve root injury, denervation degeneration, autonomic nervous system disorders, and associated complications. Thus, in the treatment of limb function recovery after SCI, both central and peripheral nerve therapies are regarded as effective [66].

Zhang et al. [67] employed a treatment regimen that combined SCs transplantation with a C5a receptor antagonist for SCI rats, finding that it significantly improved the hind limb function of the subjects. Schaal et al. [68] demonstrated through the transplantation of SCs that it can improve upper limb functions, including grip strength and self-supported forelimb suspension. These findings indicate that SCs transplantation plays a significant role in the recovery of limb function in the treatment of SCI. As previously mentioned, it has been established that the transplantation of SCs following SCI can directly inhibit inflammation and promote neural repair within the spinal cord. However, the damage to peripheral nerves resulting from SCI should not be overlooked. Wakao et al. [69] applied SCs transplantation in cynomolgus monkeys with peripheral nerve injuries and found that it had a significant therapeutic effect on limb function following these injuries. Tan et al. [70] observed the therapeutic effects of electroacupuncture (EA) stimulation of peripheral nerves in conjunction with SCs transplantation on rats with compressive SCI. They found that, compared to SCs transplantation alone, EA could enhance the survival rate of transplanted SCs, promote myelin regeneration, and reduce immune rejection reactions [70]. This inevitably leads us to propose the hypothesis that simultaneous treatment of peripheral nerves and the spinal cord may more effectively improve limb function following SCI. However, we have not identified any related studies that may involve more complex crosstalk pathways, thereby necessitating further validation and exploration.

### 2.2. SCs Transplantation Reduce the Formation of Dense Scar Tissue and Promote Neuronal Regeneration

Neurons play a fundamental role in the normal function of the spinal cord [71]. Damage to neuron axons and demyelination are direct consequences of SCI. In order to restore the normal function of the spinal cord, it is necessary to repair and regenerate neurons. However, the neurons in the CNS have limited regenerative capacity [72]. Therefore, early intervention is crucial for the effective treatment of SCI.

SCs, which are derived from neural crest cells [73], have the ability to induce neuronal regeneration in both the peripheral and CNSs [74]. A study conducted by Houmou et al. [19] demonstrated that transplantation of SCs can effectively replace or supplement damaged glial cells, leading to a significant improvement in neurological function. However, it is important to note that this glial cell repair process may also have negative effects. Several studies [75–77] have revealed that following SCI, various types of glial cells derived from the CNS (such as NG2+ oligodendrocytes [78], astrocytes, microglia, etc.), immune cells, fibroblasts, and apoptotic cell fragments [79] form dense glial scars within the injured area through complex mechanisms. These cells can form an abnormal schwannoma-like structure [80] but do not contribute to SCI restoration. Although glial scars do provide some level of protection at the site of injury, their dense spatial structure [81], the presence of inflammatory factors, and certain active molecules [82] can significantly hinder neuron regeneration and repair. Dias et al. [83] utilized Glast-CreER^T2^ transgenic mice with the ROSA26-YFP allele to decrease extracellular matrix deposition and scar formation. Their study revealed that inhibiting scar formation can reduce neuronal apoptosis and facilitate neuronal regeneration. Similarly, Assinck et al. [18] observed that transplanting skin precursor-derived SCs (SKP-SCs) into injured spinal cords effectively reduces glial scar formation and creates a favorable environment for neuron regeneration. Do-Thi et al. [84] also intervened in spinal cord-injured animals using Lv-shGFAP, which prevents glial scarring and arrived at the same conclusion. Qu et al. [85] combined the application of chondroitinase ABC (ChABC) with SCs transplantation, which better prevents the transplanted SCs from forming localized aggregates and developing into lumpy glial scars. This approach allows various repair factors to penetrate the dense barrier formed by glial scars, thereby achieving improved therapeutic outcomes. They believe this process may be related to the direct dissolution of scars, the promotion of the fusion of SCs with scar components, or the modulation of related signaling pathways [85]. Additionally, SCs transplantation can promote neuron regeneration by suppressing inflammation [86].

### 2.3. SCs Transplantation Alleviates Neurogenic Pain

After SCI, patients exhibit variations in clinical symptoms due to individual differences and the extent of the injury. However, chronic neuropathic pain [87] is a prevalent manifestation among most patients. Previous studies suggest that apoptosis and autophagy [88], mitochondrial oxidative stress [89], activation of residual spinothalamic transmission pathways [10, 90], ischemia-reperfusion, and immune factors [91] all play significant roles in causing pain.

In previous trials, intraspinal neural stem cell (NSC) transplantation therapy was found to cause abnormal neuropathic pain [92]. This may be attributed to hypersensitivity and axonal sprouting of neurons. However, Hill et al. [93] discovered that intravertebral transplantation of SCs does not lead to abnormal pain, providing further evidence for the safety of SCs transplantation. Subsequent clinical trials [20, 94, 95] have also shown that SCs transplantation can alleviate neuropathic pain in patients and improve the recovery of motor and sensory neurons to some extent. Furthermore, Hu et al. [96] found that SCs abundantly express ciliary neurotrophic factor (CNTF) and identified the CNTF-STAT3-IL-6 axis as a potential mediator of the SC-neuron-microglia neural inflammatory cascade, which could be a target for treating neuronal pain. Similarly, lysophosphatidic acid (LPA) interacts with the peripheral and CNSs to induce neuropathic pain [97], and it also promotes the proliferation and dedifferentiation of SCs [98, 99]. Therefore, it is hypothesized that the LPA-SCs pathway may be a potential target for treating neuropathic pain resulting from SCI.

### 2.4. SCs Transplantation Can Improve Urinary Dysfunction Caused by SCI

SCI not only causes motor and sensory dysfunction below the level of injury but also impairs autonomic function [100, 101]. This impairment is typically characterized by abnormalities in bladder detrusor and/or bladder sphincter reflexes [102]. Abnormal reflexes can lead to dyskinesia and progressive thickening of the bladder wall [103], worsening voiding disorders, and increasing the reliance on catheters. This significantly raises the risk of urinary tract infections [101, 104]. Therefore, it is crucial to prevent pathological thickening of the bladder wall following SCI for better patient prognosis.

Assinck et al. [18] found that although rats could resume voluntary voiding after a period of time following SCI, the thickened bladder wall did not show significant improvement. After performing CNS transplantation using SKP-SCs, not only was the movement of rats greatly improved, but bladder function and pathological thickening of the bladder wall were observed to some extent. In subsequent studies, researchers observed that the transplanted SKP-SCs not only survived at the transplantation site but also integrated into the damaged tissue, thereby providing a conducive matrix environment for tissue repair. Additionally, they found that the successful transplantation of exogenous SKP-SCs promoted the differentiation of oligodendrocyte precursor cells into SCs and significantly enhanced the activity of endogenous SCs. The role of myelin basic protein (MBP) in it has been confirmed [105]. Furthermore, Williams and Bunge [106] suggested that following the transplantation of SCs, a bridge-like structure would form at the site of SCI, providing insights into enhancing endogenous cell repair. However, May et al. [107] transplanted SKP-SCs into the injured spinal cord of immunosuppressed rats and found that the survival rate of SKP-SCs was not high, and pathological masses were found at the transplantation site. To enhance the survival rate of transplanted cells and reduce adverse reactions, they loaded SKP-SCs on hyaluronic acid-methylcellulose hydrogel (HAMC) or transparent gel modified with laminin and fibronectin-derived peptide sequences. The transplantation effect was significantly improved after treatment with HAMC. Furthermore, a clinical study revealed that the cotransplantation of autologous bone marrow mesenchymal stem cells and SCs significantly improved urodynamics in patients with SCI. During bladder filling, bladder compliance increased, and detrusor overactivity was reduced compared to the untreated condition [108]. This also indirectly demonstrates that SCs transplantation can, to some extent, improve the urinary function of patients with SCI. However, the specific mechanism by which it repairs the thickened bladder wall and urinary function has not been elucidated.

Ghosh et al. [109] conducted a study where they transplanted SCs that overexpressed polysialic acid (PSA). They discovered that unmodified SCs had limited ability to migrate outside the transplantation site and had a minimal effect on repairing the damaged spinal cord. This finding suggests that the lack of migration ability in SCs hinders their repair function. However, when PSA was overexpressed on the cell surface, the implanted SCs had an enhanced ability to bind to the axons of spinal cortical neurons and promote their regeneration. Building upon the therapeutic effect of SCs transplantation in SCI, Bunge et al. [110] found that the transplantation effect of SCs can be significantly improved when combined with olfactory ensheathing cells, neurotrophic factors, steroids, chondroitinase, or elevated cAMP levels. Additionally, Babaloo et al. [111] conducted a coculture study where SCs were cocultured with human endometrial stem cells (hEnSCs) and then transplanted into rats with SCI. They observed a greater recovery in tissue and function, suggesting that the transplantation strategy of SCs for SCI treatment still has the potential for optimization and improvement. However, further modification and innovation are necessary to determine if this method can be widely applied in the future.

## 3. Differences Between Preclinical and Clinical Trials of SCs Transplantation in SCI

As mentioned above, the transplantation of SCs has demonstrated significant therapeutic effects in animal models of SCI. However, despite its proven safety in clinical trials, scholars such as Monje have summarized the differing outcomes of SCs transplantation in animals and humans with SCI, indicating that substantial disparities still exist [112]. Based on the current body of research, it cannot be conclusively demonstrated that SCs transplantation is equally reliable in the treatment of patients with SCI.

Let us temporarily set aside the anatomical differences between animals and humans. Almost all animal experiments are conducted on artificially modeled animals. Similarly, in research on SCI treatment involving SCs transplantation, the SCI models in animals are nearly identical in terms of injury location, severity, and method. These factors are strictly controlled in the studies. However, in human SCI patients, significant variability in treatment efficacy and adverse reactions is observed in clinical trials due to heterogeneity in injury methods, severity, inflammation intensity, blood supply quality, fibrosis degree, and other individual differences [113]. Second, the presence of numerous confounding factors, along with challenges in follow-up and ethical requirements—such as the inability to use placebo treatments and blinding methods—presents significant limitations in clinical research [114].

In the course of research implementation, the transplantation of SCs as a form of cell therapy almost inevitably leads to varying degrees of rejection reactions. To minimize these adverse reactions, autologous SCs transplantation is commonly adopted in clinical practice. However, because these cells are derived from different donor nerves of various individuals, there are inherent differences in genetic composition, molecular expression, and growth kinetics. This variability complicates the evaluation of their efficacy using a single variable [115]. In both our own SCI rat model and in most reports from other researchers, female rats with shorter urethras are typically selected as study subjects due to the manifestation of urinary retention in SCI rats. This selection facilitates researchers in assisting their urination during post-modeling care. However, existing studies have indicated that estradiol may influence the course of SCI [116]. Whether this effect could be one of the confounding factors contributing to gender differences in its clinical application has yet to be substantiated by definitive evidence.

In the process of efficacy evaluation, experimental animals can typically be examined through sampling tests, molecular component analysis, and other methods in addition to functional assessments. However, such evaluations are entirely impractical in human patients. Most SCI patients undergo functional scoring and imaging to assess the effectiveness of their treatment after receiving therapy. However, during scoring, functional scores may be lower due to neuropathic pain associated with abnormal axonal regeneration caused by the cell transplantation itself [108]. Furthermore, the inherent limitations of imaging can lead to errors in evaluation [117].

In the early stages, the therapeutic effect of SCs on SCI was not well understood, and there was limited knowledge about their migration, localization, and mechanism in the body after transplantation. As mentioned earlier, clinical studies on safety and reliability indicate that the survival rate of SCs transplantation, particularly for single SCs transplantation at the site of transplantation, is low. Consequently, the cells may lose their activity before they can exert therapeutic effects. Furthermore, if their distribution cannot be artificially controlled after transplantation, SCs may form local glial clumps, which can be even more detrimental to the recovery from SCI. Additionally, due to the difficulty cells face in crossing the BBB, intravenous injection is currently rarely utilized for SCs transplantation. The potential harm to patients resulting from in situ transplantation to the damaged spinal cord cannot be overlooked. In summary, due to these various limitations, SCs transplantation has not been effectively applied in clinical practice. However, as research has progressed, it has been discovered in recent years that the effect of cell transplantation is not solely attributed to the transplanted cells themselves. Instead, it is believed that the therapeutic benefits may be attributed to the extracellular vesicles secreted by the transplanted cells. As a result, an increasing number of studies have started to investigate the therapeutic effects and mechanisms of SC-derived extracellular vesicles on SCI.

## 4. The Therapeutic Effect of Extracellular Vesicles Derived From SCs on SCI

In the nervous system, various glial cells interact closely with neurons, providing physical support, nutrition, protection, and other essential functions. The primary types of glial cells include astrocytes, oligodendrocytes, and microglia, as well as plasma cells and SCs. There are multiple mechanisms for signal transmission between glial cells and between glial cells and neurons, with extracellular vesicle communication being a significant mode of interaction. In recent years, researchers have been exploring the effects of exosomes derived from various glial cells on SCI. However, these exosomes differ significantly in terms of their cell sources and components, leading to distinct mechanisms of action. For instance, exosomes derived from glial cells serve as a notable example, and they are shown in Table 2.

The therapeutic effect of SC-exos on SCI has garnered significant attention. However, the lack of understanding regarding its mechanism has prevented any reported clinical trials. Previous studies have demonstrated that SC-exos may regulate inflammatory response [129, 130], prevent CSPG deposition [131], induce apoptosis and autophagy [132], promote angiogenesis [133], facilitate oligodendrocyte migration, and enhance neuronal axon regeneration. Furthermore, it is speculated that SC-exos may also play a role in the communication between nerve cells and glial cells, as well as between central nerves and peripheral nerves.

### 4.1. SC-Exos Participate in Immune Regulation and Reduce Inflammatory Response

The inflammatory response following SCI is highly complex and not yet fully understood. However, it is evident that the polarization of macrophages/microglia plays a crucial role in the inflammatory process [134]. Macrophages/microglia exhibit different shapes and functions, such as M1 (pro-inflammatory macrophages) and M2 (anti-inflammatory macrophages), in response to various inflammatory signals at the injury site after SCI [36]. The regulation of these two cell types at the injury site indirectly influences inflammation. Therefore, understanding macrophage/microglia polarization is of significant importance in addressing the secondary injury of SCI.

**Figure 1:**
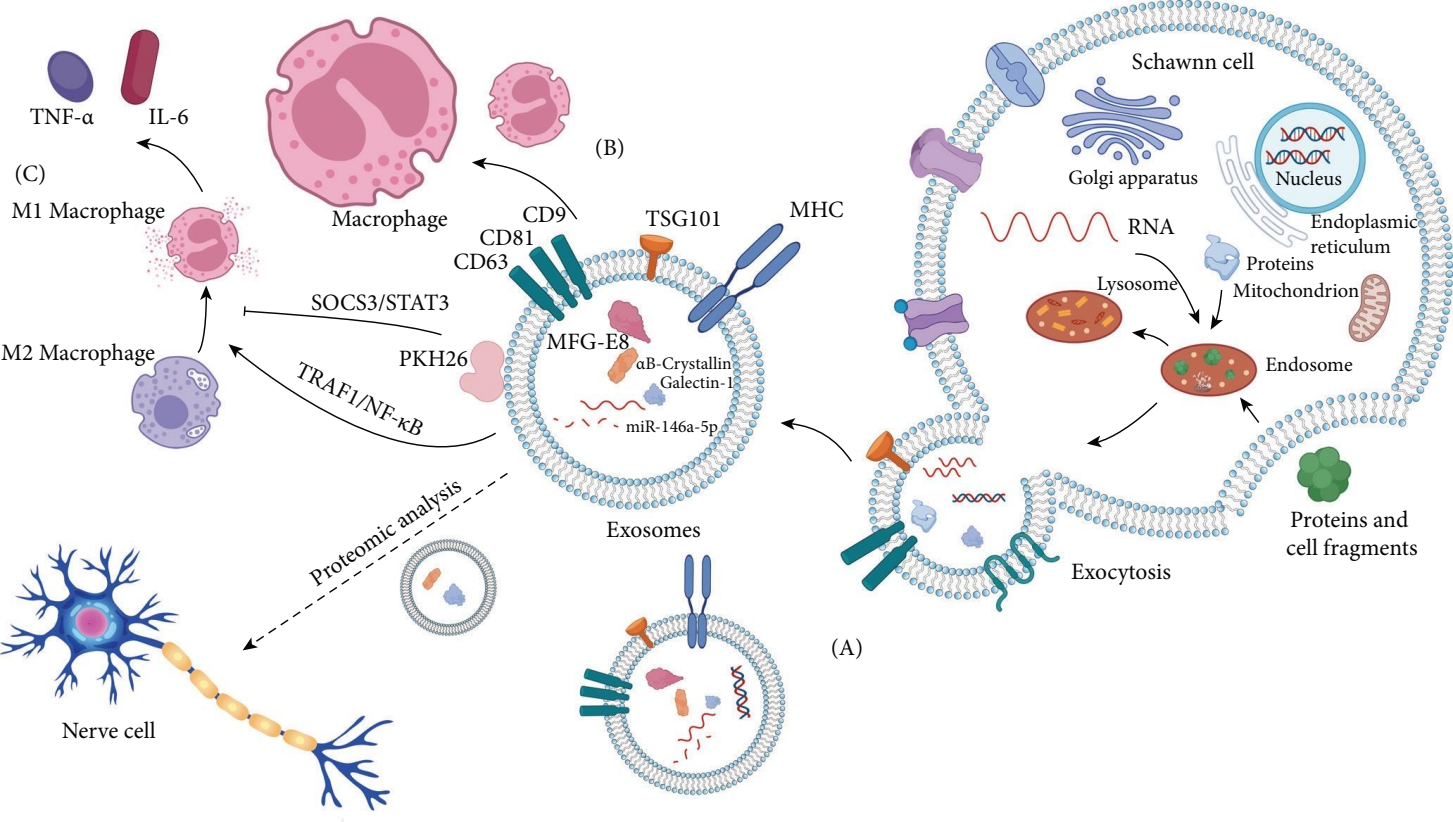
(A) To enhance clarity and conciseness, certain components of cells and exosomes are excluded from the figure, such as specific surface markers, heat shock proteins, cytoskeletal proteins, adhesion molecules, membrane transporters, fusion proteins, nucleic acids, and lipids, among others. Exosomes are derived from endosomes, which are formed when cells internalize extracellular proteins and cell debris. Intracellular materials are integrated within these endosomes to generate multivesicular bodies, which are ultimately secreted from the cell through exocytosis to produce exosomes [21]. (B) Exosomes possess low immunogenicity and good biodegradability, making them a promising option. They demonstrate a robust protective effect on biologically active substances and can efficiently traverse physiological barriers within the body, such as the blood–brain barrier, while evading immune recognition [27]. Consequently, exosomes exhibit a high level of reliability and security. (C) M1 macrophages play a role in promoting positive immune responses and perform immune surveillance functions through the secretion of pro-inflammatory cytokines and chemokines. On the other hand, M2 macrophages have limited antigen presentation ability and secrete inhibitory cytokines. Immune response is downregulated by IL-10 or TGF-B, among others.

SC-exos have the ability to cross the BSCB and transport inflammation-related content, suggesting their potential role in regulating inflammation in the CNS [135]. In a study by Ren et al. [129], PKH26-labeled SC-exos were co-cultured with bone marrow-derived macrophages, and it was observed through confocal microscopy that macrophages phagocytosed SC-exos. Subsequent animal experiments and in vitro cell experiments demonstrated that SC-exos can inhibit M1 polarization and promote M2 polarization, thereby exerting an anti-inflammatory effect. It was also discovered that this effect may be mediated by MFG-E8 carried in SC-exos, which reduces inflammation through the SOCS3/STAT3 pathway. Another study by Sun et al. [130] confirmed that SC-exos can downregulate miR-146a-5p and reduce the inhibitory effect on the TRAF1/NF-κB pathway after peripheral nerve injury. This promotes the transformation of macrophages from M2 to M1, thereby promoting the inflammatory response and hindering the recovery of SCI. These findings are consistent with the anti-inflammatory and neuroprotective effects of exosomes derived from mesenchymal stem cells on SCI, as demonstrated by Wang et al. [136] through the inhibition of NF-κB-p65 activation. Hao et al. [133] injected hydrogel loaded with SC-exos into bone defects and observed a rapid decrease in M1, a relative increase in M2, and a significant reduction in the expression of pro-inflammatory factors such as TNF-α and IL-6. Zhu et al. [137] combined SC-exos, methylprednisolone, and a nanofiber scaffold hyaluronic acid hydrogel to create a patch that demonstrated good biocompatibility, particularly in stabilizing exosome morphology and minimizing toxicity to nerve cells. Furthermore, it was verified that this composite patch facilitates the conversion of macrophages from the M1 type to the M2 type through the TLR4/NF-κB, MAPK, and Akt/mTOR pathways, thereby inhibiting the inflammatory response. Additionally, it enhances neuronal survival by reducing neuronal apoptosis following SCI. This approach is anticipated to provide a novel, noninvasive clinical treatment method for patients with SCI. Furthermore, proteomic analysis of SC-exos [138] revealed the presence of molecules, including αB-crystallin and Galectin-1, that are beneficial for CNS repair (Figure 1). These findings highlight potential targets for attenuating the inflammatory response in SCI.

### 4.2. SC-Exos Inhibiting the Deposition of CSPG and Alleviating Damage to the BSCB

Following a CNS injury, neurons have limited capacity for regeneration. To provide temporary protection, a dense glial scar, primarily composed of extracellular matrix macromolecules produced by glial cells in the CNS, including CSPG, is formed in the injured area [139] CSPG, known as an axonal growth inhibitory factor [140], plays a crucial role in hindering neuron regeneration in SCIs.

Pan et al. [131] confirmed that SC-exos can induce the expression of Toll-like receptor 2 (TLR2) on astrocytes in SCI mice through different interventions. This leads to a reduction in the deposition of CSPG. Subsequently, the TLR2 gene was knocked out, and the IKKβ inhibitor SC-514 was used to verify that SC-exos may increase TLR2 expression on astrocytes via the NF-κB/PI2K signaling pathway, resulting in reduced CSPG deposition and promoting recovery in mice. Astrocytes are currently recognized as an integral component of the BSCB [141]. Bai et al. [142] employed ChABC to enzymatically degrade CSPG at the injury site in SCI rats treated with β2-adrenoceptor agonist. They found that ChABC treatment reduced glial scarring and collagen deposition caused by CSPG. Additionally, ChABC increased the number of astrocytes involved in demyelination, contributing to the restoration of the BSCB. Wei et al. [138] identified several proteins in SC-exos related to the CNS microenvironment through proteomics and the KEGG pathway database. Notably, Rac1 and Cdc42 were found to play a crucial role in SCI recovery. As previously reported by Zheng et al. [143], Rac1 can protect the integrity of the BSCB by repairing endothelial cells, providing indirect evidence for the protective effect of SC-exos on the BSCB. Huang et al. [144] designed a scaffold with a helical structure and gradient peptide modifications, which provide essential support and space for nerve repair following SCI. This scaffold successfully loaded MSC-exos for SCI treatment, playing a significant role in the process. We believe that if the stent is equipped with SC-exos, it can not only enhance the therapeutic efficiency of the exosomes and reduce the generation of CSPG but also leverage the beneficial effects of CSPG, thereby greatly improving overall efficacy.

Goncalves et al. [145] discovered that NG2+ cells synthesize retinoic acid (RA), which is then released along with its exosomes. The RA pathway interacts with RA receptor-β (RARβ) and RA receptor-α (RARα), promoting the differentiation of oligodendrocyte precursors and remyelination. This highlights the connection between remyelination and neuron-glia crosstalk. Additionally, NG2+ cells are a significant source of CSPG secretion, suggesting that this pathway could potentially be explored for the treatment of SCI.

### 4.3. SC-Exos Induce Autophagy and Reducing Cell Apoptosis

Autophagy is a cellular process that helps maintain homeostasis by breaking down damaged cellular structures and organelles [146]. The role of autophagy and its impact on the nervous system is still debated, but recent studies suggest that autophagy provides protection in neurotrauma models [147].

Cell apoptosis, another important mechanism for maintaining internal homeostasis, is known to occur in SCIs due to various factors such as inflammation [148], oxidative stress [149], and ischemia-reperfusion [150]. Recent research has revealed a significant overlap between apoptosis and autophagy [151]. There is mounting evidence of crosstalk between these two processes [152], and Brunelli et al. [153] demonstrated that staurosporine triggers autophagy before inducing apoptosis. Additionally, the regulation of PINK1 plays a role in the transition from autophagy to apoptosis, as evidenced by the PINK1-Beclin1 interaction, highlighting the interplay between autophagy and apoptosis.

Pan et al. [132] utilized SC-exos to treat rats with SCI and observed significant improvement in motor function and reduction in neuronal apoptosis, as determined by TUNEL staining. Subsequent in vitro cell experiments demonstrated that SC-exos can enhance autophagy by increasing the expression of LC3, Beclin-1, and p62 in H_2_O_2_-induced apoptosis PC12 cells. These findings suggest that SC-exos may inhibit Akt/mTOR signaling by downregulating EGFR. Ren et al. [129] also arrived at a similar conclusion after coculturing PC12 cells with bone marrow-derived macrophages. Notably, the connection between autophagy and neuropathic pain has been reported by some scholars [154], and He et al. [155] further investigated the involvement of inflammation, apoptosis, glial scar formation, and the PI3K/AKT pathway. These studies indicate the existence of a potential common pathway or upstream mechanism in the secondary injury stages of SCI.

### 4.4. SC-Exos Promote Angiogenesis

Local vascular defects or loss caused by SCI can result in tissue ischemia and initiate an inflammatory response. Consequently, this inflammatory response hampers the natural healing process of the damaged tissue [156]. Hence, it is crucial to address vascular injury and facilitate vascular regeneration as effective approaches for SCI repair.

**Figure 2:**
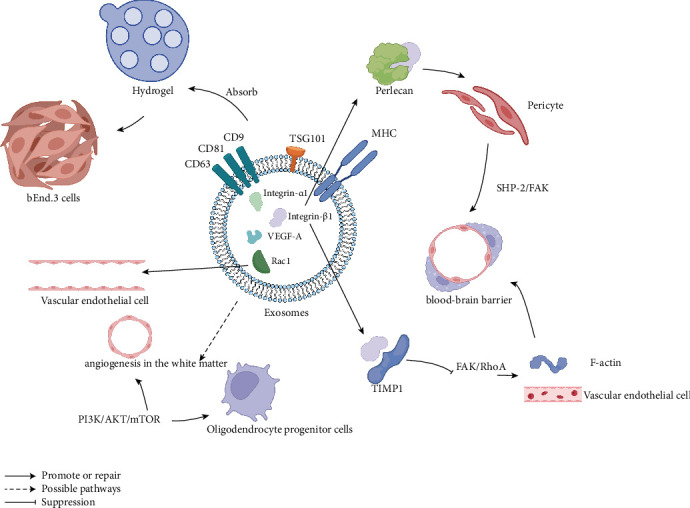
Proved or speculated pathway of promoting angiogenesis by extracellular vesicles derived from Schwann cells.

Hao et al. [133] conducted a study using hydrogel loaded with SC-exos to treat rats with bone defects. They observed a significant increase in the number and dominated area of new blood vessels in the hydrogel treatment group compared to the untreated group. In another study, Huang et al. [157] demonstrated that SC-exos can be taken up by brain-derived endothelial cells (bEnd.3 cells). Through various tests, such as the EdU test, cross-pore chamber migration assay, and capillary network formation test, they showed that SC-exos have an influence on endothelial cells and promote vascular regeneration. Further investigation revealed high expression of integrin-β1 and integrin-α1, as well as relatively low expression of VEGF-A, in SC-exos. These molecules are likely to play a role in vascular regeneration following SCI. Previous research has indicated that the combination of integrin-β1 and Perlecan can enhance angiogenesis and repair the BBB through SHP-2/FAK-mediated pericyte migration [158]. In the context of traumatic brain injury, the interaction between TIMP1 and integrin-β1 plays a role in inhibiting the FAK/RhoA pathway. This inhibition leads to F-actin depolymerization and stabilization of the vascular endothelial structure, ultimately promoting the functional recovery of the BBB [159]. Additionally, it has been mentioned earlier that Rac1 expressed in SC-exos can contribute to the repair of endothelial cells. Wang et al. [160] discovered that in the early stages of PI3K/AKT/mTOR pathway activation, there was a significant increase in the proliferation of oligodendrocyte progenitor cells and angiogenesis in the white matter. The general mechanism is shown in Figure 2. However, further research is needed to determine whether there is a causal relationship between SC-exos and these effects.

### 4.5. SC-Exos May Promote Differentiation and Migration of Oligodendrocytes, Repair Myelin Sheath

In the white matter of the CNS, oligodendrocytes wrap myelinated nerve fibers to form myelin. This myelin serves various functions, including electrical insulation, mechanical protection, metabolic nutrition, and regulation of communication [161]. While SCs and oligodendrocytes have different origins and distribution, their differentiation pathways and regulation of neurons are remarkably similar [162]. The connection between the CNS and PNS can be established through multiple signaling pathways [163].

After SCI, neurons are continuously damaged due to defects and loss of myelin. Oligodendrocyte progenitor cells can quickly respond by proliferating and differentiating into new oligodendrocytes [164], thereby generating new myelin sheaths [165]. oligodendrocyte progenitor cells are derived from NSCs [162]. Glial scars, primarily composed of CSPGs, are formed by glial cells compensatorily after nerve injury, severely inhibiting nerve function. Galindo et al. [166] discovered that the glycosaminoglycan side chain CS of CSPG may inhibit the migration of NSCs to the injury site through the RhoA/ROCK signaling pathway. A similar view was proposed by Contreras et al. [167]. Additionally, SC-exos can effectively reduce the expression of CSPG and clear deposited CSPG by activating autophagy. Zhu et al. [168] found that after injecting SC-exos into SCI mice, the deposition of CSPG in scar tissue at the injury site was reduced, while the expression of the CSPG-specific receptor protein tyrosine phosphatase-sigma (PTP-σ) increased as axons grew around the injury site. This increase in expression correlates with a significant reduction in CSPG production, which facilitates spinal cord recovery. Additionally, the use of Rho/ROCK inhibitors was shown to inhibit the reparative effects of SC-exos on scar tissue following SCI. Therefore, it is concluded that SC-exos induce axon growth after SCI by decreasing PTP-σ activation on CSPG via the Rho/ROCK pathway, thus expanding our understanding of this mechanism. Furthermore, Dong et al. [169] concluded that the Rac1/Cdc42 signaling pathway can promote the migration of oligodendrocytes, and Rac1 is highly expressed in SC-exos. Therefore, it is speculated that SC-exos can indirectly promote the migration of NSCs, oligodendrocyte progenitor cells, and oligodendrocytes to the injury site for myelin repair. Li et al. [170] also observed enhanced proliferation and differentiation of NSCs when cocultured with SCs, which was attributed to the secretion of neurotrophic factors by SCs. The KEGG analysis of the SC-exos proteome [138] revealed that the expression of neurotrophic factor-related signaling pathways was significantly higher (*p*  < 0.05). This suggests that SC-exos may also play a role in the differentiation of NSCs.

Furthermore, it has been observed that SC-exos may contain myelin protein (papaya-like protease [PLP]) [127]. PLP has been found to have a unique advantage in repairing damaged myelin sheaths in the CNS [17]. As a result, it is necessary to further investigate the potential role of SC-exos in promoting NSC differentiation and oligodendrocyte migration.

### 4.6. SC-Exos May Promote Axonal Regeneration of Spinal Cord Neurons

Neuronal axon destruction and impairment of regeneration are significant aspects of SCI [171]. The prevention of further axon destruction and the promotion of axon regeneration are crucial for the treatment of SCI. In a model of skull injury, hydrogels loaded with SC-exos demonstrated a greater ability to promote neuronal regeneration [133]. As mentioned earlier, SCs possess a strong reprograming ability following SCI, where differentiated SCs (dSCs) transform into repair-type SCs (rSCs) [51]. These rSCs then initiate endogenous sexual repair pathways to play a reparative role. López-López-Leal et al. [128] were the first to confirm that the reprograming of SCs in peripheral nerve injury is accompanied by the secretion of exosomes. This is evidenced by the expression of c-Jun and Sox2 on SCs, as well as the expression of miRNA-21 in SC-exos. The downregulation of PTEN and activation of PI3 kinase have shown to promote axonal regeneration. Unfortunately, the therapeutic effect of SC-exos on SCI through the same or similar mechanism has not been confirmed. However, similar pathways have been identified in the repair pathway of SCI [172–174]. Moreover, exosomes themselves have a strong ability to cross the BSCB. Therefore, we speculate that SC-exos can promote axonal regeneration of spinal cord neurons by crossing the BSCB and employing a mechanism similar to that observed in peripheral nerve injury.

In addition, SC-exos have been shown to regulate immunity, inhibit inflammation, reduce apoptosis, and regulate the microenvironment of the CNS. These effects themselves have a certain neuroprotective effect, suggesting that SC-exos may indirectly promote axonal regeneration of spinal cord neurons.

### 4.7. SC-Exos May Regulate Communication Between CNS Cells and Glial Cells

In the early days, when the concept of glial cells was first proposed, they were initially believed to exist independently of neurons and only play a “passive” role, such as mechanical protection. However, increasing evidence suggests that glial cells are not only regulated by neurons but also have endocrine functions that regulate the activities of neurons [175]. Following the confirmation that glial cell transplantation promotes myelination and neuron regeneration, many scholars have started investigating its mechanism. Research indicates that glial cells not only enter neurons by secreting paracrine signaling molecules like nucleotides and excitatory amino acids, but they also transport specific proteins or RNA through extracellular vesicles for directional or semi-directional communication with neurons in order to achieve regulation [176, 177].

Lopez-Verrilli and Court [178] have discovered that SCs, similar to oligodendrocytes and microglia, can secrete exosomes and deliver elongation factors necessary for mRNA translation. Their study also showed that SC-exos can promote peripheral nerve axonal regeneration [179]. Although the role of SCs in the CNS has not been fully confirmed, subsequent reports have proposed some mechanisms. For example, it has been suggested that SCs inhibit GTPase Rho to prevent growth cone collapse [135] and that P75^NTR^ regulates growth cone filopodia [179]. Additionally, SCs can alter the expression of related genes and indirectly transmit signaling molecules, such as cytokines, chemokines, pro-regenerative factors, and neuronal growth regulatory factors, to neurons through exosomes [180]. These findings provide evidence of close communication between glial cells and neurons.

Berkowitz et al. [181] discovered that neuroinflammation in the CNS and PNS may communicate with each other through the complement system and coagulation system. Dezawa [182] proposed that the environment of the PNS not only influences the PNS itself but also stimulates regeneration in the CNS. Suter and Jaworski [183] suggested that, under certain influences, specific cells can selectively traverse the boundary between the CNS and PNS, thereby connecting the two nervous systems. These findings strongly indicate that the CNS and PNS are not entirely independent systems. SC-exos, as a means of bridging the gap between the two, may have the potential to facilitate crosstalk between nerve cells, glial cells, and the CNS-PNS pathways, making it a promising avenue for SCI treatment.

## 5. Current Research Trends and Gaps in the Field

Recent research on SCs and SC-exos in treating SCIs has revealed their mechanisms of action. These studies have shown both innovative approaches and limitations, as summarized in Tables 3 and 4.

Research trends have evolved from SCs transplantation to SC-exos, transitioning from in vitro cell experiments to in vivo animal studies, and from focusing on epiphenomenon to cellular molecular mechanisms. As experimental designs and sample sizes improve, research on omics and new materials is increasingly prevalent. Despite these advancements, the lack of attention from researchers has hindered a comprehensive understanding of the molecular mechanisms and raised concerns about safety, thus preventing the qualification for large-scale clinical trials.

The incorporation of SCs with PSA not only enhances cell migration and axon regeneration capabilities but also allows for tailored modifications to meet individual patient requirements. For instance, in cases of complete spinal cord rupture, optimizing the ratio and combination of SCs and PSA enables the design of more precise treatment plans for optimal nerve repair outcomes. Furthermore, coculturing SCs with human intimal stem cells enhances the safety and efficacy of cell therapy by replicating the natural growth environment of autologous cells. This approach holds promise for patients with extensive nerve damage from surgery or trauma, as it enables the reconstruction of intricate neural networks crucial for restoring motor and sensory functions. Additionally, the use of PCL/gelatin nanofiber scaffolds offers a biodegradable and versatile platform to support and guide SCs at the injury site. These scaffolds can be customized based on specific injury conditions, allowing for adjustments in shape, size, and mechanical properties to accommodate different types and severities of nerve damage. The application of exosomes is an innovative strategy that allows for treatment without directly using living cells, instead encapsulating and protecting bioactive molecules like growth factors, nucleic acids, and proteins. This approach is particularly significant in clinical settings as it can potentially reduce immune rejection and other complications from cell transplantation while also aiding in nerve repair and regeneration. Given the intricate blend of biology, engineering, ethics, and regulatory considerations involved in the clinical translation of these technologies, future advancements will necessitate interdisciplinary collaboration and careful management in both preclinical and clinical trials to guarantee the safety and efficacy of these treatments. Moreover, as research delves deeper into the molecular mechanisms of stem cells, a more thorough understanding will further propel the translation of these technologies into clinical practice, offering enhanced treatment options for patients with neurological injuries.

Although initial clinical studies have shown the safety and potential efficacy of SCs in treating nerve injuries, there are some key limitations. Many studies had small sample sizes and lacked diverse patient populations, limiting the generalizability and statistical significance of the findings. Future research should focus on larger randomized controlled trials with diverse patient populations in terms of age, gender, injury type, and severity. Additionally, existing research mainly examines short-term effects, lacking evaluation of long-term effects and potential side effects. Extended follow-up studies are necessary to assess treatment durability and long-term safety. Further research is needed to enhance the long-term functional performance of transplanted cells and optimize cell transplantation technology for improved stability and therapeutic effects. These improvements will allow for a more comprehensive evaluation of the potential and limitations of SCs transplantation in nerve injury treatment, providing a stronger scientific foundation for future clinical applications.

Based on our review and conjecture, future research should focus on elucidating the complex molecular mechanisms through which SCs contribute to SCI repair and mitigate bladder wall thickening. This research may require the utilization of gene editing and molecular labeling techniques to track SCs' movements, localization, and differentiation in vivo, along with advancements in tissue engineering to improve transplantation vectors and enhance SC survival and repair efficiency in injured spinal regions. Additionally, a quantitative analysis of the interaction between SC-induced glial scarring and neural regeneration is essential, utilizing high-throughput sequencing and proteomics to explore the molecular composition of SC-exos, with a specific focus on key molecules such as *α*B-Crystallin, Galectin-1, Rac1, and Cdc42 in CNS recovery. Furthermore, a detailed investigation into the neuroinflammatory processes involving the CNTF-STAT3-IL-6 axis among SCs, neurons, and microglia is crucial for understanding its role in neuropathic pain and evaluating the therapeutic potential of the LPA-SCs pathway for SCI-induced neuropathic pain. The involvement of NG2+ cells in CSPG secretion and their therapeutic implications in SCI also warrant exploration. Cellular-level studies using in vitro cultures and animal models should examine the impact of the PI3K/AKT and mTOR pathways on oligodendrocyte proliferation, angiogenesis, and their interactions with SC-exos following SCI. These comprehensive investigations aim to address knowledge gaps in SCI repair and establish a strong scientific basis for innovative therapeutic approaches.

## 6. Future Perspectives and Conclusion

SCs and their derived exosomes provide a promising avenue for potential therapeutic strategies in patients with SCIs. To further advance this field, future research should focus on exploring the following aspects:

• Safety and Efficacy Verification: Establishing the safety and efficacy of SC-exos therapy necessitates further clinical trials. Addressing immune rejection, excessive proliferation, and other adverse reactions is paramount. Research focusing on molecular modifications and innovative materials could enhance transplantation success and safety, warranting prioritization before clinical application.
• Mechanism Research Enhancement: Deepening our understanding of SCs and their exosomes is essential. Before discussing the differences in therapeutic effects between SCs transplantation and their exosomes, it is essential to thoroughly investigate their mechanisms of action. For instance, it remains unclear whether the effects of SCs transplantation are mediated solely through their exosomes, through a combination of exosomes and other mechanisms, or if the exosomal pathway is the primary mechanism underlying the therapeutic effects. Therefore, in subsequent studies, Rab27a in SCs can be knocked down, or SCs can be treated with GW4869 to block exosome production to fully explore their mode of action. Studies demonstrating the potential for SCI treatment through direct communication and interaction within damaged areas underscore the need for exploring unknown mechanisms. Advancements in multiomics and molecular docking could unveil novel therapeutic pathways, improving research outcomes in SCI.
• Optimizing Treatment Strategy: Effective treatment strategies necessitate precise optimization of timing, periodicity, and dosage to adequately balance the benefits of treatment against the risks of inflammation and scarring. SC transplantation may be more appropriate as a complementary intervention, as its effects tend to diminish over time. In contrast, SC-exos hold significant promise for drug delivery, particularly due to their ability to traverse the BBB. To further refine treatment plans, it is essential to carefully consider factors such as source selection, purification methods, and administration techniques. Enhancing these strategies through cell and exosome engineering could improve targeting and long-term efficacy. Furthermore, investigating alternative routes of administration beyond intravenous injection in future studies may enhance therapeutic effectiveness.
• Multidisciplinary Integration and Transformation: Animal experiments and clinical experience indicate that achieving optimal therapeutic effects with a single drug or treatment method can be challenging. In contrast, combined applications may yield synergistic effects, exemplified by the principle of “one plus one is greater than two.” Therefore, a multimaterial and multidisciplinary approach may be essential for the clinical translation of SC-exos in the treatment of SCI.

In summary, the use of SCs and their exosomes has shown potential in improving SCI through their direct effects on CNS-PNS crosstalk and neuron-glia crosstalk. Based on current research results, SCs, and their exosomes have been found to have potential in treating SCI through various mechanisms. First, they can regulate immune cell activity and reduce inflammation, which helps protect damaged spinal cord tissue. Second, they can protect the BSCB. Third, they contain nerve regeneration factors that promote neuron regeneration and protect neurons from apoptosis. Fourth, they can improve the local microenvironment by promoting vascular regeneration, leading to improved neurological function. Lastly, they can regulate communication between CNS cells and glial cells, inhibiting glial scar formation and promoting myelin regeneration.

## Figures and Tables

**Table 1:** The role of Schwann cells in different situations.

Characteristics and functions	Specific role	Reference
Physiological condition		
Forming myelin sheath	Increase axonal electrical signal conduction speed	[41]
	Regulating intra-axonal transport	[42, 43]
	Neurotrophic effects	[44, 45]
Cell regulation	Regulating neuronal excitability	[46]
	Affects the migration of stem cells from the spinal cord	[47]
	Regulating the morphology and function of peripheral nerves	[48]
Differentiation function	Has the potential to differentiate into other glial cells	[49]
Pathological condition		
Nerve repair	Reprograming and dedifferentiation after axonal interruption	[50, 51]
	Inducing autophagy of glial cells and immune cells	[52, 53]
	Change the morphology of astrocytes	[54]
Cancer spread	Secretory paracrine signaling molecules	[55, 56]
	Scattered cancer cells after Schwann cell recombination	[57]
Immunoregulation	Interactions with immune cells	[58–60]

**Table 2:** Cargoes carried by extracellular vesicles produced by different glial cells under different stimuli and their effects.

Source of exosomes	Intervene	Cargo	Effect	Reference
Astrocyte	IL-1β, TNF-α	miR-125a-5p, miR-16-5p	Reduce the entanglement of neuronal dendritic processes	[118]
	Ethanol	TLR4, NF-κb-p65, IL-1R, caspasa-1, NLRP3	Cascade amplification of neuronal inflammatory response	[119]
	—	Synapsin	Stimulating the growth of neural processes	[120]

Oligodendrocyte	—	PLP, MOG, MAG, CNP	Inhibition of oligodendrocyte differentiation and myelin formation	[121]
	OGD	SOD, catalase	Improve metabolic rate under oxidative stress and reduce neuronal apoptosis	[122]

Microglia	—	Aminopeptidase CD13, IDE	The former has not yet reported its effectiveness, but it can be used to detect the secretion of extracellular vesicles. The latter can help degrade the extracellular matrix β amyloid protein	[123, 124]
	IL-4	miR-124, miR-137	Exhibiting neuroprotective effects under conditions of oxygen–glucose deprivation	[125, 126]

Schwann cells	Prion infection	PLP, CNP, MBP, PrPc, PrPsc	Related to prion infection of the central nervous system	[127]
	Peripheral nerve injury	miR-21	Promote the transformation of differentiated SCs into reparative SCs, promoting neural repair	[128]

**Table 3:** The main animal experiments involved in this article.

Title	Year	Research object	Innovation	Boundedness	Reference
Restoration of normal conduction properties in demyelinated spinal cord axons in the adult rat by transplantation of exogenous Schwann cells	1996	Rats	They found that SCs can low repulsively myelinate the CNS	No evidence has been found that transplanting Schwann cells alone can lead to myelin formation	[19]
Transplantation of preconditioned Schwann cells following hemisection spinal cord injury	2007	Rats (*n* = 24)	Peripheral nerve grafts can promote axonal formation	Small sample size and short observation time	[106]
Graft of preinjured sural nerve promotes regeneration of corticospinal tract and functional recovery in rats with chronic spinal cord injury	2008	Rats (*n* = 54)	Proved that peripheral nerve grafts can promote axonal regeneration and are related to SCs	There is no evidence to suggest that a large number of transplanted SCs actively migrate to the uninjured host's CNS	[107]
Dissociated predegenerated peripheral nerve transplants for spinal cord injury repair: a comprehensive assessment of their effects on regeneration and functional recovery compared to Schwann cell transplants	2012	Rats (*n* = 63)	Dissociated predegenerated nerves transplantation cannot provide sufficient improvement for SCI repair	No purified SCs were tested	[105]
Extensive cell migration, axon regeneration, and improved function with polysialic acid-modified Schwann cells after spinal cord injury	2012	Rats (*n* = 34)	SCs modified with PSA can improve SCI through cell migration and axonal regeneration	Unclear relationship between PSA, SCs, and SCI	[112]
Adult skin-derived precursor Schwann cell grafts form growths in the injured spinal cord of Fischer rats	2018	Rats (*n* = 20)	Cultivating SKP-SCs in modified hyaluronic acid methylcellulose can improve the survival rate of transplantation in SCI	Failed to solve the problem of excessive proliferation of SCs	[84]
Schwann cell transplantation subdues the pro-inflammatory innate immune cell response after spinal cord injury	2018	Rats	SCs can inhibit the immune response of SCI	The role of SCs transplantation in CNS is not as effective as PNS	[98]
Schwann cell-like differentiated adipose stem cells promote neurite outgrowth via secreted exosomes and RNA transfer	2018	Extracorporeal neural cells	SC-exos can promote the growth of neurons in vitro	No animal in vivo experiments conducted	[121]
Proteomics analysis of Schwann cell-derived exosomes: a novel therapeutic strategy for central nervous system injury	2019	—	Neurotrophic factors, PI3K-Akt, and cAMP signaling pathways in SC exos play important roles in CNS repair	No experimental verification conducted	[123]
PCL/gelatin nanofibrous scaffolds with human endometrial stem cells/Schwann cells facilitate axon regeneration in spinal cord injury	2019	Rats	Cocultivation of hEnSCs and SCs can enhance their ability to regenerate axons	Its safety should be further verified	[114]
CNTF-STAT3-IL-6 axis mediates neuroinflammatory cascade across Schwann cell-neuron-microglia	2020	Mice	SCs may treat neuropathic pain in SCI through CNTF-STAT3-IL-6 therapy	How CNTF affects the CNS microenvironment has not been elucidated	[108]
Transplantation of skin precursor-derived Schwann cells yields better locomotor outcomes and reduces bladder pathology in rats with chronic spinal cord injury	2020	Rats (*n* = 47)	SCs can improve SCI and prevent pathological thickening of the bladder	Its safety needs further verification	[18]
Increasing Toll-like receptor 2 on astrocytes induced by Schwann cell-derived exosomes promotes recovery by inhibiting CSPGs deposition after spinal cord injury	2021	Mice (*n* = 90)	SCs can promote recovery by inhibiting CSPGs deposition after SCI	Is there a risk of neurofibroid transformation that has not been elucidated	[117]
Autophagy induced by Schwann cell-derived exosomes promotes recovery after spinal cord injury in rats	2021	Rats (*n* = 120)	SCs can improve SCI by enhancing autophagy and reducing apoptosis	Its molecular mechanism has not been elucidated	[118]
Schwann cells-derived exosomes promote functional recovery after spinal cord injury by promoting angiogenesis	2023	Rats (*n* = 40)	SCs-exos delivers integrins-β1 promote angiogenesis	It is not the only cause of angiogenesis	[141]
Schwann cell-derived exosomes containing MFG-E8 modify macrophage/microglial polarization for attenuating inflammation via the SOCS3/STAT3 pathway after spinal cord injury	2023	Rats (*n* = 200)	A large sample size has been used to demonstrate that SC-exos can reduce inflammatory response	Not paying attention to its impact on nerve regeneration	[115]

**Table 4:** Clinical trials currently completed by SCs.

Title	Year	Research object	Results	Conclusion	Reference
Treatment of chronic thoracic spinal cord injury patients with autologous Schwann cell transplantation: an interim report on safety considerations and possible outcomes	2008	Human with chronic thoracic SCI (*n* = 4)	Only one subject showed significant improvement in movement and sensation	Safety and effectiveness should be further determined	[63]
A prospective randomized double-blind clinical trial using a combination of olfactory ensheathing cells and Schwann cells for the treatment of chronic complete spinal cord injuries	2014	Human with chronic complete SCI (*n* = 28)	Except for one subject, transplantation of olfactory ensheathing cells (OECs) and/or SCs can improve SCI	Transplanting OECs, SCs, or their combination has good tolerance and is beneficial to patients	[184]
Safety of autologous human Schwann cell transplantation in subacute thoracic spinal cord injury	2017	Human with subacute thoracic SCI (*n* = 6)	No adverse reactions or progression of the condition	Highly purified autologous SCs for spinal transplantation are feasible	[185]
Phase 1 safety trial of autologous human Schwann cell transplantation in chronic spinal cord injury	2022	Human with chronic SCI (*n* = 8)	One subject showed significant improvement in all aspects, while the other subjects also received varying degrees of relief, but the effect weakened over time, and no adverse reactions were observed	Proved the feasibility and safety of SCs in treating chronic SCI participants	[113]
Scalable culture techniques to generate large numbers of purified human Schwann cells for clinical trials in human spinal cord and peripheral nerve injuries	2022	Human with acute SCI (*n* = 7), human with chronic SCI (*n* = 8), and human with peripheral nerve injuries (*n* = 3)	Prove that transplanted cells can retain vitality and have certain safety	This provides a relatively safe and effective manufacturing method for autologous transplantation of SCs products	[186]

## Data Availability

Data sharing is not applicable to this article as no datasets were generated or analyzed during the current study.

## Nomenclature

TLR4: Toll-like receptor 4
NF-κb-p65: Nuclear factors that activate B cells κ light chain enhancer
IL-1: Interleukin-1
NLRP3: NOD-like receptor protein 3
PLP: Papaya-like protease
MOG: Myelin oligodendrocyte glycoprotein
MAG: Myelin-associated glycoprotein
CNP: C-type natriuretic peptide
OGD: Oxygen and glucose deprivation
SOD: Superoxide dismutase
IDE: Insulin degrading enzyme
MBP: Myelin basic protein
PrP: Prion protein
PrPc: Cell-type prion proteins
PrPsc: Cell-type prion proteins isomers
TNF-α: Tumor necrosis factor-α
LPA: Lysophosphatidic acid
SOCS3: Suppressor of cytokine signaling 3
STAT3: Signal transducer and activator of transcription 3
TRAF1: Receptor-associated factor-1
PINK1: PTEN-induced putative kinase 1
VEGF-A: Vascular endothelial growth factor A
SHP-2: Src homology 2 domain-containing protein tyrosine phosphatase 2
FAK: Focal adhesion kinase
TIMP1: Tissue inhibitor of matrix metalloprotease 1
mTOR: Mammalian target of rapamycin
ROCK: RhoA/Rho kinase
PTEN: Phosphatase and tensin homolog.
